# Loop-ileostomy reversal—patient-related characteristics influencing time to closure

**DOI:** 10.1007/s00384-018-2994-x

**Published:** 2018-03-05

**Authors:** Carl Pontus Gustafsson, Ulf Gunnarsson, Ursula Dahlstrand, Ulrik Lindforss

**Affiliations:** 1 Department of Surgery, Visby Hospital, Gotland, Sweden; 2 Department of Molecular Medicine and Surgery, Karolinska Institutet, Stockholm, Sweden; 3 Department of Surgical and Perioperative Science, Umeå University, Umeå, Sweden; 4 Department of Clinical Sciences, Intervention and Technology (CLINTEC), Karolinska Institutet, and Centre for Digestive Diseases, Karolinska University Hospital, Stockholm, Sweden; 5 Department of Molecular Medicine and Surgery, Karolinska Institutet, and Centre for Digestive Diseases, Karolinska University Hospital, Solna, Sweden

**Keywords:** Defunctioning stoma, Loop-ileostomy, Reversal, Socioeconomic factors, Low anterior resection

## Abstract

**Purpose:**

To identify factors associated with timing of stoma reversal after rectal cancer surgery in a large Swedish register-based cohort.

**Methods:**

Three thousand five hundred sixty-four patients with rectal cancer who received a protective stoma during surgery in 2007–2013 were identified in the Swedish colorectal cancer register. Time to stoma reversal was evaluated over a follow-up period of one and a half years. Factors associated with timing of stoma reversal were analysed using Cox regression analysis. Reversal within 9 months (12 months if adjuvant chemotherapy) was considered latest expected time to closure.

**Results:**

Stoma reversal was performed in 2954 (82.9%) patients during follow-up. Patients with post-secondary education had an increased chance for early stoma reversal (HR 1.13; 95% CI 1.02–1.25). Postoperative complications (0.67; 0.62–0.73), adjuvant chemotherapy (0.63; 0.57–0.69), more advanced cancer stage (stage III 0.74; 0.66–0.83 and stage IV 0.38; 0.32–0.46) and higher ASA score (0.80; 0.71–0.90 for ASA 3–4) were associated with longer time to reversal. Two thousand four hundred thirty-seven (68.4%) patients had stoma reversal within latest expected time to closure. Factors associated to decreased chance of timely reversal were more advanced cancer stage (stage III 0.64; 0.50–0.81 and stage IV 0.19; 0.13–0.27), postoperative complications (0.50; 0.42–0.59) and higher ASA score (0.77; 0.61–0.96 for ASA 3–4).

**Conclusions:**

Patients with a high level of education had a higher chance of timely reversal but medical factors had a stronger association to time to reversal. Patients with advanced rectal cancer are at high risk for non-reversal and should be considered for permanent stoma.

## Introduction

Rectal cancer operated with low anterior resection has an anastomotic leakage rate of 2–28% [1–3]. A nationwide Swedish study indicated that temporary faecal diversion with a defunctioning loop-ileostomy reduces the consequences of anastomotic leakage after low anterior resection in rectal cancer surgery [2]. This and other reports [1] resulted in increasing numbers of patients with a loop-ileostomy after low anterior resection for rectal cancer [4]. The morbidity risk associated with loop-ileostomy includes leakage from the stoma dressing, skin problems and dehydration, as well as temporary or chronic renal failure [5–7]. Temporary stoma has a negative impact on patient quality-of-life (QoL). QoL improves after reversal [8], but in some cases, problems remain such as temporary alteration in bowel function, often with additional social and economic burdens [9]. Delay in reversal of the ileostomy may be associated with an increased risk for complications [10]. Early reversal 8–14 days after primary surgery is feasible and has been claimed to be safe [11, 12]. Despite this, most clinics report a delay of between 3 and 6 months or even longer before reversal. The period between creation and reversal should be kept as short as possible, and performed after nutritional optimisation of the patient. Such necessary steps also affect the time between the index operation and stoma reversal. Stoma reversal is associated with several complications with overall morbidity rates of up to 45% [7, 13, 14]. In 21–28% of cases, defunctioning stomas are not reversed, becoming permanent [10, 15, 16]. Reasons for not reversing a stoma include advanced cancer stage and anastomotic leakage [16]. Temporary loop-ileostomy can safely be reversed in due time, and in most countries, this occurs within 3 months, though there are several reasons why reversal should be performed as soon as possible, as mentioned above. In some cases, the interval between primary surgery and reversal is prolonged. Common reasons for delayed closure are ongoing adjuvant chemotherapy or surgical complications. Another reason could be shortage of staff creating a queue, with other operations being given priority [16].

Socioeconomic factors have been shown to be associated with stoma reversal rate [15, 17, 18]. Income, education and occupation are well-known variables used for classification of socioeconomic status [19, 20]. Kuryba et al. [15] evaluated factors affecting stoma reversal rate in 4879 rectal cancer patients. They found an association between socioeconomic deprivation and low reversal rate.

Studies analysing risk factors for delayed stoma reversal contra permanent stoma after rectal cancer surgery are often small, and only a few studies have analysed the association between socioeconomic factors and stoma reversal rate. Most studies have focused on stoma reversal contra permanent stoma.

The aim of this study was to identify factors determining the timing of stoma reversal after rectal cancer surgery in a large Swedish register-based cohort.

## Method

The study population was retrieved from the Swedish Colon and Rectal Cancer Register (SCRCR). The study included all patients with rectal cancer operated on by anterior resection with a defunctioning stoma between 1 January 2007 and 31 December 2013.

Since 1995, all patients diagnosed with an adenocarcinoma of the rectum in Sweden are registered in the SCRCR. Data are reported to the register by the surgeons and pathologists involved and the register’s national coverage is 99% [21]. The register has been validated and shown to be of high quality [22].

Data from the SCRCR include information on patient characteristics, preoperative workup, procedural details (including whether a defunctioning stoma was created), tumour characteristics, complications, planned oncologic therapy, effected oncologic treatment and follow-up results (e.g. local recurrence, metastases, late complications, stoma reversal, death). Since January 2011, postoperative complications are not only registered according to type, but also classified according to the Clavien-Dindo classification; this information is not available for procedures prior to 2011. Data were retrieved from the SCRCR on 12 May 2016.

To confirm that information regarding date of stoma reversal was complete, data from the Swedish National Patient Register (NPR) were also retrieved. Since 1987, the NPR includes data on all inpatient care in Sweden, and it is mandatory for Swedish health providers (county councils) to report all inpatient data to the NPR. Coverage of the NPR register has been estimated to be more than 99% and validity is high [23]. All data on inpatient care of patients in the study population including records of stoma reversal (using the Swedish Classification of Surgical and Medical Procedures) were obtained.

Socioeconomic data on income and level of education at the time of cancer surgery were obtained from Statistics Sweden, the administrative agency responsible for developing, producing and distributing official statistics and other governmental statistics. Statistics Sweden also provided data on if and when patients in the study group had emigrated or died.

The outcomes investigated were reversal of stoma within a latest expected time to closure (yes/no) and time to reversal of stoma. “Latest expected time to closure” was defined as within 9 months of stoma creation in patients who did not receive adjuvant chemotherapy, and 1 year from stoma creation in patients who were planned for adjuvant chemotherapy. The time limit of 9 months was chosen considering a median time to closure of over 6 months. Ongoing adjuvant chemotherapy is usually a contraindication to stoma reversal; therefore, the time span allowed for patients who received adjuvant chemotherapy was extended for 3 months (and the limit set to 1 year). Time to reversal of stoma was evaluated over a follow-up period of one and a half years following creation of stoma.

### Statistical analyses

Statistical analyses were performed using Stata/SE 12.1 (StataCorp, College Station, TX, USA). Uni- and multivariable logistic regression analyses were performed to detect factors associated with reversal of the loop-ileostomy during the study period. Factors assessed were age, sex, ASA physical status classification, cancer stage according to the TNM classification system, postoperative complication (yes/no), adjuvant chemotherapy, low economic standard (disposable income below 60% of the median income of the entire population; yes/no) and level of education (primary or lower secondary education/upper secondary education/post-secondary education). Possibly, determining factors for the multivariable analysis were selected based on univariable analysis and on hypothesised relevance. A multivariable Cox proportional hazard regression model was used to estimate the hazard rates regarding time from the construction to the reversal of the stoma for the investigated factors. The same factors as in the logistic regression were investigated. Possibly, determining factors for the multivariable analysis were selected based on univariable analysis.

## Results

### Descriptive data

A total of 3564 patients who received a protective stoma during their primary rectal cancer operation (i.e. low anterior resection) between January 1, 2007 and December 31, 2013 were identified. The median age at the time of creation of the stoma was 66 years (range 23–90). Of these, 2177 (61.1%) patients were male and 1387 (38.9%) were female. Patient characteristics are accounted for in Table 1 and Table 2 presents frequency and type of complications following the cancer operation.

**Table 1:** Patient characteristics and stoma reversal data for 3564 patients who had rectal cancer surgery with a diverting loop-ileostomy in 2007–2013

Median age, years (range)	66 (23–90)
Male, no. (%)	2177 (61.1)
ASA score, no. (%)	
1 2 3–4	898 (25.2) 2079 (58.3) 530 (14.9)
Cancer stage, no. (%)	
0–1 2 3 4	998 (28.0) 997 (28.0) 1228 (34.5) 249 (7.0)
Low economic standard, no. (%)	589 (16.5)
Level of education, no. (%)	
Lower secondary Upper secondary Post-secondary	1005 (28.2) 1369 (38.4) 883 (24.8)
Adjuvant chemotherapy, no. (%)	1213 (34.0)
Stomas reversed within 1.5 years, no. (%)	2954 (82.9%)
Median time to reversal, days (range)	191 (5–458)

**Table 2:** Postoperative complications necessitating treatment in 3564 patients who had rectal cancer surgery with a diverting loop-ileostomy in 2007–2013. Severity of complication according to the Clavien-Dindo classification

	Infectious, *n* (%)	Cardiovascular, *n* (%)	Neurologic, *n* (%)	Surgical, *n* (%)	Other, *n* (%)	Total, *n* (%)^†^
Treated complication	213 (6.0)	119 (3.3)	8 (0.2)	793 (22.3)	456 (12.8)	1406 (39.5)
Clavien-Dindo class						
2	97 (2.7)	34 (1.0)	5 (0.1)	108 (3.0)	129 (3.6)	292 (8.2)
3a	4 (0.1)	2 (0.1)	2 (0.1)	93 (2.6)	9 (0.3)	100 (2.8)
3b	4 (0.1)	1 (0)	–	109 (3.1)	15 (0.4)	115 (3.2)
4a	9 (0.3)	2 (0.1)	–	3 (0.1)	6 (0.2)	18 (0.5)
4b	2 (0.1)	–	–	–	–	2 (0.1)
5	2 (0.1)	4 (0.1)	1 (0)	–	7 (0.2)	13 (0.4)
Clavien-Dindo missing*	95 (2.7)	76 (2.1)	–	480 (13.5)	290 (8.1)	866 (24.3)

*Patients with reported complication, but without registered Clavien-Dindo score. Clavien-Dindo was only registered for the 1660 patients who had the operation in 2011–2013

Reversal of the stoma was performed in 2954 (82.9%) patients during the follow-up period. Median time to reversal in these patients was 191 days (range 5–458 days). The stoma was reversed within stipulated latest expected time to closure (12 months if the patient had adjuvant chemotherapy, otherwise 9 months) in 2437 (68.4%) patients. One hundred and fifty-two patients died during the follow-up without having had their stoma reversed; no one was lost to follow-up due to emigration.

### Stoma reversal within latest expected time to closure—logistic regression

Associations between the factors analysed and stoma reversal within latest expected time to closure or not are presented in Table 3. A decreased chance for timely stoma reversal was seen for patients who had postoperative complications after primary cancer surgery (odds ratio [OR] 0.50, 95% confidence interval [CI] 0.42–0.59) and for patients with cancer stages 3 or 4 according to UICC (OR 0.64, 95% CI 0.50–0.81 and OR 0.19, 95% CI 0.13–0.27, respectively). Delayed reversal or non-reversal was more likely in patients with a higher ASA class at the time of cancer surgery (OR for reversal of stoma 1.26, 95% CI 1.03–1.53 for ASA 1; OR 0.77, 95% CI 0.61–0.96 for ASA 3–4). No association between rate of stoma reversal and age, sex or level of income was seen. Patients whose highest level of education was post-secondary had an increased odds ratio for stoma reversal within latest expected time to closure, but the difference was not statistically significant (OR 1.24, 95% CI 1.00–1.55).

**Table 3:** Odds ratio (OR) for stoma reversal within “latest expected time to closure”* in 3564 patients who had rectal cancer surgery with a diverting loop-ileostomy in 2007–2013

	Stoma reversed	Univariable model		Multivariable model	
	*n* (%)	OR (95% CI)	*p*	OR (95% CI)	*p*
Age					
0–65 years	1143/1620 (70.6)	1 (ref)			
66 years	1294/1944 (66.6)	0.83 (0.72–0.96)	0.011	0.92 (0.77–1.09)	0.33
Sex					
Female	976/1387 (70.4)	1 (ref)			
Male	1461/2177 (67.1)	0.86 (0.74–0.99)	0.042	0.95 (0.80–1.13)	0.57
ASA score					
1	673/898 (74.9)	1.42 (1.19–1.70)	< 0.001	1.26 (1.03–1.53)	0.024
2	1409/2079 (67.8)	1 (ref)			
3–4	312/530 (58.9)	0.68 (0.56–0.83)	< 0.001	0.77 (0.61–0.96)	0.021
Cancer stage					
0–1	755/998 (75.7)	1 (ref)			
2 3 4	726/997 (72.8) 795/1228 (64.7) 90/249 (36.1)	0.86 (0.71–1.05) 0.59 (0.49–0.71) 0.18 (0.14–0.24)	0.15 < 0.001 < 0.001	0.93 (0.75–1.16) 0.64 (0.50–0.81) 0.19 (0.13–0.27)	0.53 < 0.001 < 0.001
Postop compl.					
No	1612/2156 (74.8)	1 (ref)			
Yes	824/1406 (58.6)	0.48 (0.41–0.55)	< 0.001	0.50 (0.42–0.59)	< 0.001
Adjuvant chemo					
No	1645/2288 (71.9)	1 (ref)			
Yes	768/1213 (63.3)	0.67 (0.58–0.78)	< 0.001	0.87 (0.71–1.07)	0.20
Low economic standard**					
No	2054/2975 (69.0)	1(ref)			
Yes	383/589 (65.0)	0.83 (0.69–1.00)	0.056	1.05 (0.82–1.33)	0.72
Level of education					
Lower secondary	681/1005 (67.8)	1 (ref)			
Upper secondary Post-secondary	950/1369 (69.4) 650/883 (73.6)	1.08 (0.91–1.29) 1.33 (1.09–1.62)	0.40 0.005	1.02 (0.84–1.23) 1.24 (1.00–1.55)	0.84 0.051

*“latest expected time to closure” time: within 1 year of stoma creation in patients who had adjuvant chemotherapy, within 9 months of stoma creation in patients without adjuvant chemotherapy

**Low economic standard implies a disposable income lower than 60% of the median value in the population

### Factors influencing time to stoma reversal—Cox proportional hazards regression

**Fig. 1:**
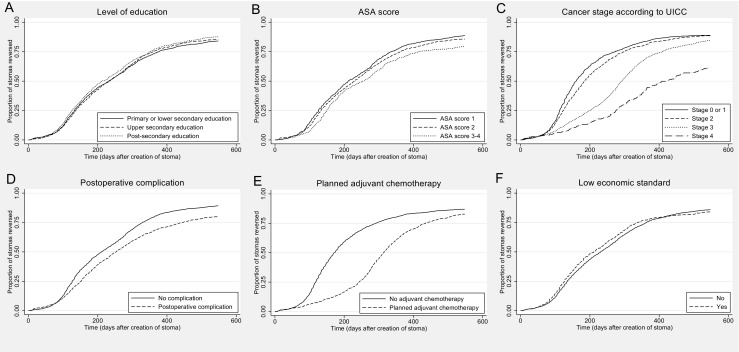
**a**–**f**. Kaplan-Maier curves for the cumulative proportion of patients who have had their stoma reversed. **a** Level of education. **b** ASA physical status classification. **c** Cancer stage according to UICC. **d** Postoperative complication. **e** Planned adjuvant chemotherapy. **f** Low economic standard

Table 4 accounts for the findings in the Cox proportional hazards analysis. In the multivariable analysis, an association with longer time to stoma reversal was seen for patients with postoperative complications after primary surgery (hazard ratio [HR] 0.67, 95% CI 0.62–0.73) and patients planned for adjuvant chemotherapy (HR 0.63, 95% CI 0.57–0.69). Patients with cancer stage 3 (HR 0.74, 95% CI 0.66–0.83) or cancer stage 4 (HR 0.38, 95% CI 0.32–0.46) according to UICC had later stoma reversal, just as patients with higher ASA class (HR 1.14, 95% CI 0.1.04–1.24 for ASA class 1; ASA class 2 reference; and HR 0.80, 95% CI 0.71–0.90 for ASA classes 3–4). Patients with post-secondary education had an increased chance for early stoma reversal (HR 1.13, 95% CI 1.02–1.25). Figure 1a–f contains the Kaplan-Meier plots for cumulative proportion of patients whose stoma was reversed for the preoperatively known predictor variables included in the multivariable analyses.

**Table 4:** Hazard ratio (HR) for stoma reversal within 1.5 years in 3564 patients who had rectal cancer surgery with a diverting loop-ileostomy in 2007–2013

	Univariable model		Multivariable model	
	HR (95% CI)	*p*	HR (95% CI)	*p*
Age				
0–65 years	1 (ref)			
66 years	1.03 (0.96–1.11)	0.45		
Sex				
Female	1			
Male	0.94 (0.87–1.01)	0.11		
ASA score				
1	1.10 (1.01–1.20)	0.022	1.14 (1.04–1.24)	0.005
2	1		1	
3–4	0.83 (0.75–0.93)	0.001	0.80 (0.71–0.90)	< 0.001
Cancer stage				
0–1	1		1	
2 3 4	0.89 (0.81–0.97) 0.57 (0.52–0.63) 0.31 (0.26–0.37)	0.012 < 0.001 < 0.001	0.96 (0.87–1.06) 0.74 (0.66–0.83) 0.38 (0.32–0.46)	0.45 < 0.001 < 0.001
Adjuvant chemotherapy				
No	1		1	
Yes	0.56 (0.52–0.60)	< 0.001	0.63 (0.57–0.69)	< 0.001
Complication				
No	1		1	
Yes	0.74 (0.68–0.79)	< 0.001	0.67 (0.62–0.73)	< 0.001
Low economic standard				
No	1		1	
Yes	1.04 (0.94–1.15)	0.44	1.02 (0.91–1.15)	0.71
Level of education				
Lower secondary	1		1	
Upper secondary Post-secondary	1.03 (0.94–1.12) 1.11 (1.01–1.22)	0.58 0.038	1.02 (0.93–1.12) 1.13 (1.02–1.25)	0.65 0.023

*Low economic standard implies a disposable income lower than 60% of the median value in the population

## Discussion and conclusions

In this study, the median time to loop-ileostomy reversal was approximately 6 months which is far longer than it should be, and the time recommended. Although creation of a defunctioning loop-ileostomy is considered to be a safe means of diversion, there are complicating factors associated with the procedure. The stoma itself causes physical and emotional trauma and also additional economic burden for the patient. The interval between creation and closure is often prolonged and some loop-ileostomies are never reversed. Complications after closure can also have a severely negative impact on patient quality-of-life. There is a need for clear guidelines regarding the procedure and timing of reversal. Some studies have shown that patients who recover quickly after surgery should have their stoma closed as soon as possible in order to avoid stoma-associated problems such as dehydration and renal dysfunction [24] as well as reduce the cost to society and to the patient. After closure of a defunctioning stoma, the majority of patients experience an improvement in the quality of their life, and this is more pronounced when the stoma is closed within 3 months [25]. In spite of this, patients continue to have complaints at follow-up after stoma closure [9]. In the present study, about 32% of loop-ileostomies created at the primary operation were not reversed within latest expected time to closure. Many patients who had their stoma closed during the follow-up (88%) had to wait more than 3 months i.e. the interval between surgery for rectal cancer and stoma reversal was substantially longer than is usually anticipated. Ongoing adjuvant chemotherapy is usually a contraindication to stoma reversal and this treatment may last for 6 months followed by fatigue and sometimes malnutrition.

The term “latest expected time to closure” was introduced to allow a partition for whether a specific stoma reversal was considered to be delayed (in relation to what would be expected in this population) or not, and defined by ourselves. In this population, 88% of the reversals were performed more than 3 months after the creation of the stoma and the median time to closure was over 6 months. We defined the latest expected time to stoma closure to be within 9 months in patients who were not treated with adjuvant chemotherapy.

In this study, postoperative complications and chemotherapy were shown to delay reversal of the stoma, as could be anticipated. We could not demonstrate an association between income and time to stoma reversal, but patients with a high level of education were found to have a higher chance of timely reversal. One possible explanation for this association is that patients living in urban areas that are closer to hospital facilities may also have a higher level of education, although the data at hand does not allow any firm conclusions to be drawn.

In this study, reversal of the stoma was performed in 82.9% patients during the follow-up period. Compared to other studies [26, 27], this is a normal to high reversal rate. Medical records were not reviewed and the available data in this study does not offer information on the reason for the stoma becoming permanent in the specific cases of non-reversal.

Stoma reversal is delayed in almost 90% of patients. It is important to plan for early reversal and stoma reversal operations should be given priority. The patient should preoperatively be informed about the “true” expected time to reversal, rather than about the recommended or desired time to reversal. Patients who have risk factors for permanent stoma must be given the opportunity from the start to choose permanent colostomy at their operation as this will improve their chances of leading a good quality life with a stoma. Surgeons and healthcare providers must be aware that socioeconomic factors are risk factors for delayed reversal. Sharing decision-making with the patient is a key factor when planning rectal cancer surgery.

This study on factors that possibly have an impact on the interval between primary surgery and stoma reversal was based on a large population-based patient cohort containing 3564 patients with a long follow-up period, which facilitates generalisability and interpretation of the results. Data were collected from three different registers and the patients were well-defined as a group. Socioeconomic data are registered on an individual level by Statistics Sweden. However, there are also limitations. This is a retrospective study and the validity and methods of reporting data might have differed between hospitals, even though data were retrieved from the validated SCRCR and NPR registers. Data in these registers are registered prospectively and have been shown to be of high quality. The patient group was homogenous in terms of how surgery was performed i.e. low anterior resection for rectal cancer, and the recommendation was strong, in most cases, to proceed with a temporary defunctioning stoma when creating a low colorectal anastomosis. Despite this, patient selection and other circumstances such as comorbidity could have been a possible source of bias. The most important socioeconomic determinant for the interval between creation of the loop-ileostomy and its closure was level of education. We are aware of the fact that there is no precise universally accepted definition as to what is “low” for these factors, but considered these variables to be the ones easiest to define and sub-classify, and the most reliable. The intention in all the studied patients was to create a temporary defunctioning stoma at their rectal cancer operation. But in many cases, the date planned for closure was postponed and in other cases a reversal was never performed. This particular problem must not be underestimated, and there is an urgent need for reassessment of the factors influencing the time elapsing between primary surgery and stoma reversal.

Patients with a high level of education had a higher chance of timely reversal but medical factors had a stronger association to time to reversal. Patients with advanced rectal cancer are at high risk for non-reversal and should be considered for permanent stoma.
